# ﻿Three new fold-winged crane flies of the genus *Ptychoptera* Meigen, 1803 (Diptera, Ptychopteridae) from southern China

**DOI:** 10.3897/zookeys.1122.86483

**Published:** 2022-09-28

**Authors:** Zehui Kang, Gang Gao, Xiao Zhang, Ding Yang, Junping Wang

**Affiliations:** 1 Shandong Engineering Research Center for Environment-Friendly Agricultural Pest Management, College of Plant Health and Medicine, Qingdao Agricultural University, Qingdao 266109, China Qingdao Agricultural University Qingdao China; 2 College of Plant Protection, China Agricultural University, Beijing 100193, China China Agricultural University Beijing China

**Keywords:** New species, Ptychopterinae, taxonomy

## Abstract

Three new *Ptychoptera* Meigen, 1803 species from southern China, *P.hekouensis***sp. nov.**, *P.longa***sp. nov.**, and *P.xiaohuangshana***sp. nov.**, are described and illustrated. These new species are mainly distinguished from congeners by their body colors and male genitalia. The genus *Ptychoptera* is recorded from Guangdong, China for the first time. An updated key to all Chinese *Ptychoptera* species is provided.

## ﻿Introduction

The family Ptychopteridae, also known as fold-winged crane flies, is a group of slender tipulid-like flies in the lower Diptera. Ptychopteridae are divided into subfamilies Bittacomorphinae and Ptychopterinae. The genus *Ptychoptera* Meigen, 1803 is the only extant genus in subfamily Ptychopterinae and can be easily distinguished by the antennae in adults with 13 flagellomeres, the wing with M_1+2_ forked, and the gonopod with a simple gonocoxite (Alexander 1981; Rozkošný 1997; Nakamura and Saigusa 2009).

Nineteen *Ptychoptera* species have been known to occur in China. Charles P. Alexander described three *Ptychoptera* species from China during 1924–1937 (Alexander 1924, 1935, 1937). More than half a century later, three new Chinese species were published by Yang and Chen (1995, 1998) and Yang (1996). More recently, 13 species were added to the fauna of China by Kang et al. (2013, 2019), Zhang and Kang (2021), and Shao and Kang (2021).

In the present paper, three *Ptychoptera* species from southern China – *P.hekouensis* sp. nov. from Yunnan Province, *P.longa* sp. nov. from Guizhou Province, and *P.xiaohuangshana* sp. nov. from Guangdong Province – are described and illustrated, recording the genus *Ptychoptera* for the first time from Guangdong. In addition, the key by Zhang and Kang (2021) is updated to include all Chinese species of *Ptychoptera*.

## ﻿Materials and methods

Adults were collected by entomological net and kept in 75% alcohol. Type specimens are deposited in the Entomological Museum of China Agricultural University, Beijing, China (CAU). Photographs were taken by a Canon EOS-90D and EF 100 mm f/2.8L IS USM. Genitalia were prepared by immersing the apical portion of the abdomen in warm lactic acid for 0.5–1 h. Afterwards, they were examined and illustrations prepared by using a ZEISS Stemi 2000-C stereomicroscope. After examination, the removed abdomen was transferred to fresh glycerine and stored in a microvial pinned to the respective specimen. Morphological terminology is based primarily on McAlpine (1981) and Fasbender (2014).

## ﻿Taxonomy

### ﻿Check list of Chinese *Ptychoptera* species

*Ptychopterabannaensis* Kang, Yao & Yang, 2013 (Yunnan)

*Ptychopterabellula* Alexander, 1937 (Jiangxi, Zhejiang)

*Ptychopteracircinans* Kang, Xue & Zhang, 2019 (Fujian)

*Ptychopteraclitellaria* Alexander, 1935 (Sichuan)

*Ptychopteracordata* Zhang & Kang, 2021 (Yunnan)

*Ptychopteraemeica* Kang, Xue & Zhang, 2019 (Sichuan)

*Ptychopteraformosensis* Alexander, 1924 (Taiwan; Japan)

*Ptychopteragutianshana* Yang & Chen, 1995 (Zhejiang)

*Ptychopterahekouensis* sp. nov. (Yunnan)

*Ptychopteralii* Kang, Yao & Yang, 2013 (Guizhou)

*Ptychopteralonga* sp. nov. (Guizhou)

*Ptychopteralongwangshana* Yang & Chen, 1998 (Zhejiang)

*Ptychopteralucida* Kang, Xue & Zhang, 2019 (Xinjiang)

*Ptychopteralushuiensis* Kang, Yao & Yang, 2013 (Yunnan)

*Ptychopteraqinggouensis* Kang, Yao & Yang, 2013 (Neimenggu)

*Ptychopteraseparata* Kang, Xue & Zhang, 2019 (Xizang)

*Ptychopteratianmushana* Shao & Kang, 2021 (Zhejiang)

*Ptychopterawangae* Kang, Yao & Yang, 2013 (Yunnan)

*Ptychopteraxiaohuangshana* sp. nov. (Guangdong)

*Ptychopteraxinglongshana* Yang, 1996 (Gansu)

*Ptychopterayankovskiana* Alexander, 1945 (Neimenggu; Korea)

*Ptychopterayunnanica* Zhang & Kang, 2021 (Yunnan)

### ﻿Updated key to *Ptychoptera* from China

**Table:** 

1	Wing with r-m arising from R_4+5_ after Rs fork, Rs not longer than r-m	**2**
–	ing with r-m arising from Rs before or at Rs fork, Rs at least 1.5 times length of r-m (Fig. 2a–c)	**10**
2	Mesopleuron mostly brown (Fig. 1c, e); epandrial clasper brown	***P.circinans***
–	Mesopleuron uniformly yellow (Fig. 1a); epandrial clasper uniformly yellow	**3**
3	Gonostylus long and slender, about 1.5 times length of gonocoxite	***P.bannaensis***
–	Gonostylus short, as long as gonocoxite (Fig. 4b, h)	**4**
4	Postnotum dark brown with a large yellow spot	**5**
–	Postnotum uniformly black	**6**
5	Wing with spots at forks of R_1+2_, R_4+5_ and M_1+2_ forming a band (Fig. 2c); abdomen with first tergum yellow with caudal 1/5 light brown; subapical spine of epandrium absent (Fig. 2b); anterior lobe of basal lobe of gonostylus not bilobate, medial lobe of basal lobe of gonostylus not bilobate; apical process of paramere semilunar, apex expanding outward	***P.cordata***
–	Wing with spots at forks of R_1+2_, R_4+5_ and M_1+2_ separated (Fig. 2b); abdomen with first tergum dark brown with basal 1/5 yellow; subapical spine of epandrium transverse conical; anterior lobe of basal lobe of gonostylus bilobate, medial lobe of basal lobe of gonostylus bilobate; apical process of paramere hook-shaped, apex incurvated	***P.yunnanica***
6	Wing with a distinct spot at fork of R_4+5_, spots at forks of R_1+2_ and M_1+2_ weak and nearly invisible	***P.lii***
–	Wing with three distinct spots at forks of R_1+2_, R_4+5_ and M_1+2_, separated or forming a band	**7**
7	Second tergum anterior margin yellow with a median brown spot; medial lobe of basal lobe of gonostylus slender, finger-shaped	***P.lushuiensis***
–	Second tergum anterior margin yellow brown; medial lobe of basal lobe of gonostylus board, tongue-shaped	**8**
8	Abdomen with 5^th^ and 6^th^ terga mostly yellow, 6^th^ and 7^th^ sterna yellow; apical stylus of gonostylus finger-shaped (Nakamura and Saigusa 2009)	***P.formosensis***
–	Abdomen with 5^th^ and 6^th^ terga dark brown, 6^th^ and 7^th^ sterna mostly brown; apical stylus of gonostylus hook-shaped	**9**
9	Sixth and 7^th^ sterna yellow, tip of surstylus curved up when viewed from the lateral side, retrose basal projection on inner side with tip bilobate, paramere with a pair of hook-shaped projections and a pair of conical projections, subapical sclerite of aedeagus serrated with five teeth	***P.tianmushana***
–	Sixth and 7^th^ sterna mostly brown, tip of surstylus not curved up when viewed from the lateral side, retrose basal projection on inner side not bilobate at tip, paramere with a pair of slender L-shaped projections, subapical sclerite of aedeagus serrated with two teeth	***P.emeica***
10	Mesopleuron uniformly yellow	**11**
–	Mesopleuron mostly brown or black	**14**
11	Wing with bands and clouds (Fig. 2b, c)	**13**
–	Wing without band or cloud	**12**
12	Scutellum uniformly yellow brown; 2^nd^ tergum mostly yellow with posterior margin brown; epandrial clasper without papillary projection on inner side; medial lobe of basal lobe of gonostylus semicircular	***P.wangae***
–	Scutellum mostly brownish black, middle area yellow (Fig. 1b); 2^nd^ tergum mostly brownish black with middle area yellow; epandrial clasper with two papillary projections on inner side (Fig. 4a); medial lobe of basal lobe of gonostylus ear-shaped (Fig. 4b)	***P.hekouensis* sp.nov.**
13	Base of Rs with an elliptic cloud; abdomen with sterna yellow	***P.qinggouensis***
–	Base of Rs without cloud; abdomen with sterna black (Alexander 1935)	***P.clitellaria***
14	Epandrial lobes merged with epandrial claspers (Fig. 4a, g)	**15**
–	Epandrial lobes not merged with epandrial claspers (Fig. 4d)	**20**
15	Wing with r-m separated from fork of Rs by longer than its own length; epandrial claspers short and blunt	***P.separata***
–	Wing with r-m close to fork of Rs; epandrial claspers slender	**16**
16	Wing with an elliptic cloud at middle of CuA_1_ (Fig. 2c)	**17**
–	Wing without an elliptic cloud at middle of CuA_1_	**19**
17	Epandrial clasper without a curved finger-shaped projection interiorly	**18**
–	Epandrial clasper with a curved finger-shaped projection interiorly (Fig. 4g)	***P.xiaohuangshana* sp. nov.**
18	Epandrial claspers curved downward, tip bifurcated	***P.gutianshana***
–	Epandrial claspers straight, tip not bifurcated	***P.bellula***
19	Gonostylus much longer than gonocoxite	***P.xinglongshana***
–	Gonostylus not longer than gonocoxite	***P.longwangshana***
20	Epandrium bilobed, epandrial claspers not merged basally	**21**
–	Epandrium not bilobed, epandrial claspers merged basally (Fig. 4d)	***P.longa* sp. nov.**
21	Abdomen with 2^nd^ and 3^rd^ terga brownish black; epandrial claspers finger-shaped and broad basally, curved inwards at middle	***P.lucida***
–	Abdomen with 2^nd^ and 3^rd^ terga mostly yellow; epandrial claspers flat and acinaciform, middle of inner edge slightly swollen	***P.yankovskiana***

#### ﻿ Ptychoptera hekouensis

Taxon classificationAnimaliaDipteraPtychopteridae

Kang, Gao & Zhang sp. nov.

F44654F2-32D4-5A11-A806-07D87D52E45A

https://zoobank.org/97105AE4-40AD-46BC-A51F-1E5A7E48A464

**Figure 1.**
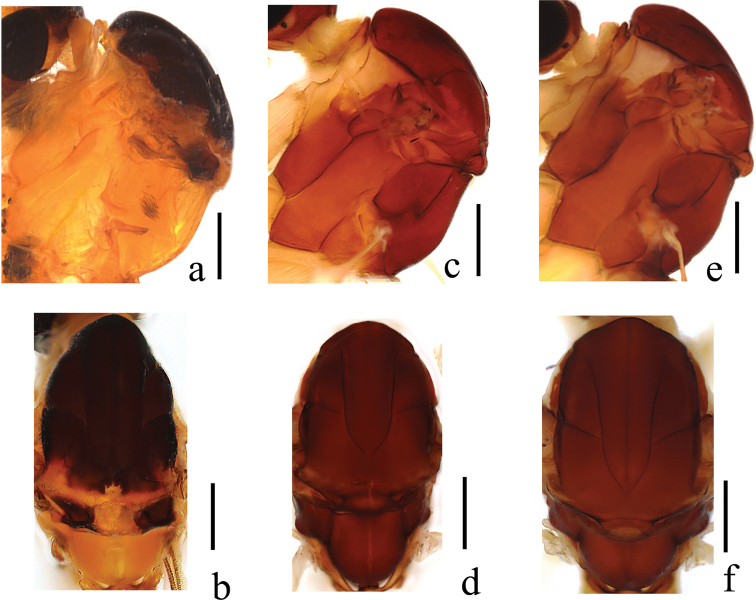
Thoraxes of new *Ptychoptera* species **a***P.hekouensis*, dorsal view **b***P.hekouensis*, lateral view **c***P.longa*, dorsal view **d***P.longa*, lateral view **e***P.xiaohuangshana*, dorsal view **f***P.xiaohuangshana*, lateral view. Scale bars: 0.5 mm.

**Figure 2.**
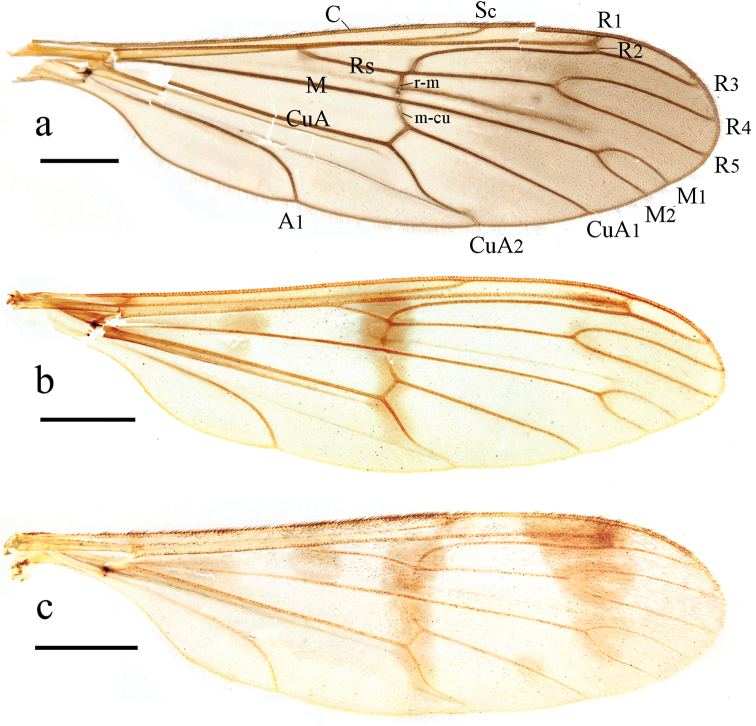
Wings of new *Ptychoptera* species **a***P.hekouensis***b***P.longa***c***P.xiaohuangshana*. Scale bars: 1.0 mm.

**Figure 3.**
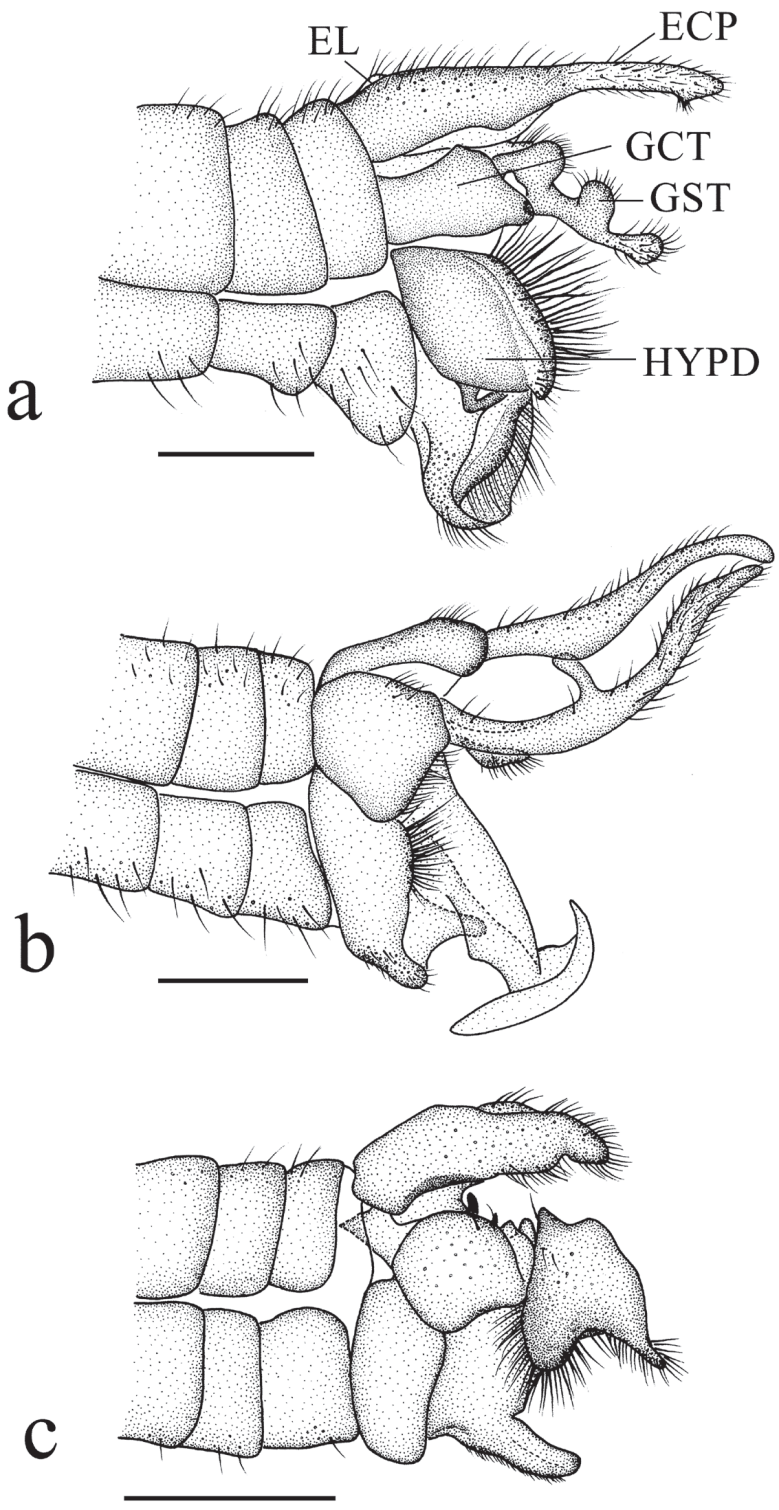
Male genitalia of new *Ptychoptera* species **a***P.hekouensis***b***P.longa***c***P.xiaohuangshana*. Scale bars: 0.5 mm. (ECP = epandrial clasper, EL = epandrial lobe, GCT = gonocoxite, GST = gonostylus, HYPD = hypandrium).

**Figure 4.**
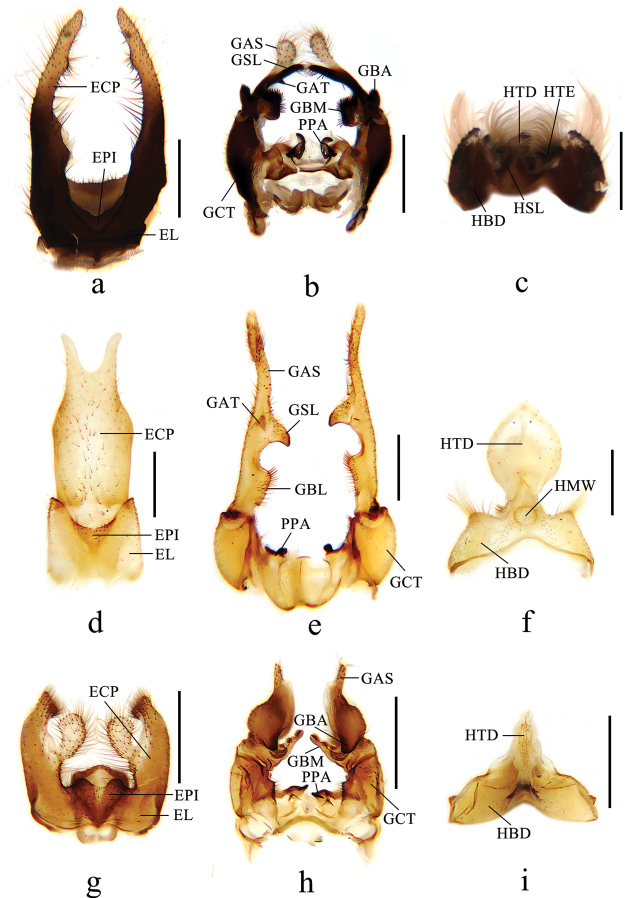
Details of male genitalia of new *Ptychoptera* species **a** epandrium of *P.hekouensis*, dorsal view **b** gonocoxite and gonostylus of *P.hekouensis*, dorsal view **c** hypandrium of *P.hekouensis*, ventral view **d** epandrium of *P.longa*, dorsal view **e** gonocoxite and gonostylus of *P.longa*, dorsal view **f** hypandrium of *P.longa*, ventral view **g** epandrium of *P.xiaohuangshana*, dorsal view **h** gonocoxite and gonostylus of *P.xiaohuangshana*, dorsal view **i** hypandrium of *P.xiaohuangshana*, ventral view. Scale bars: 0.4 mm. (ECP = epandrial clasper, EL = epandrial lobe, EPI = epiproct, GAS = apical stylus of gonostylus, GBA = anterior lobe of basal lobe of gonostylus, GBL = basal lobe of gonostylus, GBM = medial lobe of basal lobe of gonostylus, GCT = gonocoxite, GSL = secondary lobe of apical stylus of gonostylus, GAT = tertiary lobe of apical stylus of gonostylus, HBD = basal division of hypandrium, HMW = membranous window of terminal division of hypandrium, HSL = spathate lobe of hypandrium, HTD = terminal division of hypandrium, HTE = lateral extension of terminal division of hypandrium, PPA = apical process of paramere).

**Figure 5.**
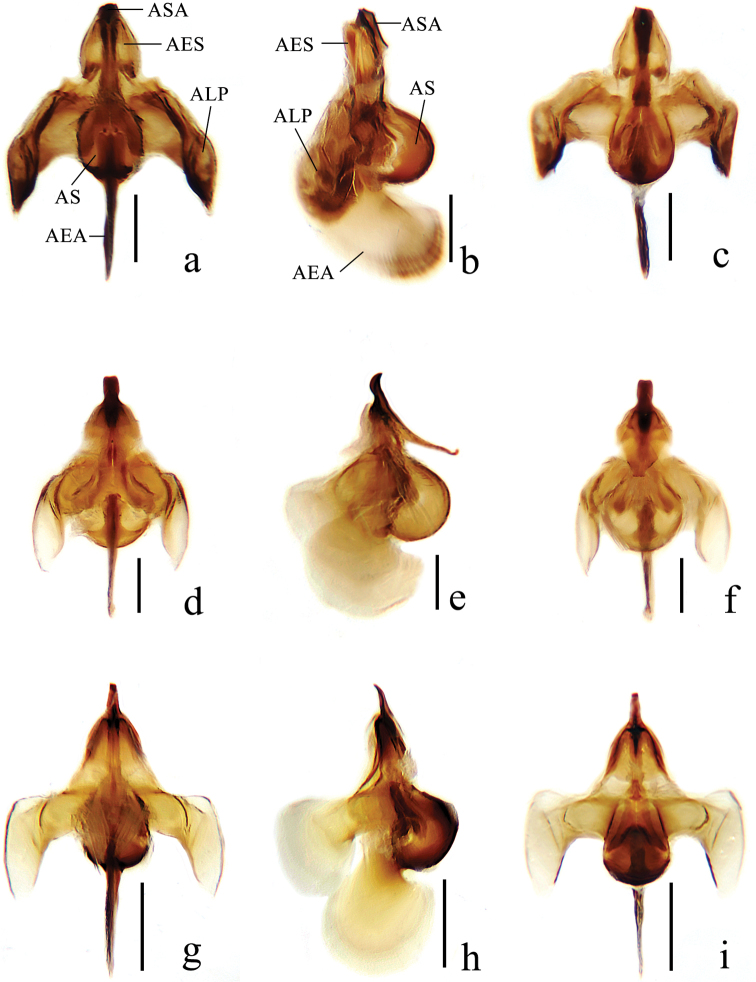
Aedeagi of new *Ptychoptera* species **a***P.hekouensis*, dorsal view **b***P.hekouensis*, lateral view **c***P.hekouensis*, ventral view **d***P.longa*, dorsal view **e***P.longa*, lateral view **f***P.longa*, ventral view **g***P.xiaohuangshana*, dorsal view **h***P.xiaohuangshana*, lateral view **i***P.xiaohuangshana*, ventral view. Scale bars: 0.2 mm. (AEA = ejaculatory apodeme, AES = aedeagal sclerite, ALP = lateral ejaculatory process, AS = sperm sac, ASA = subapical sclerite of aedeagus).

Figs 1a, b , 2a , 3a , 4a–c , 5a–c

##### Diagnosis.

Scutellum mostly brownish black, middle area yellow; wing marked with small brown marks at base of Rs, tip of R_1_, base of R_2+3_, fork of R_4+5_, r-m, and fork of M_1+2_; epandrial clasper tapering and slightly curved distally to the middle, inner side with two papillary projections; medial lobe of basal lobe of gonostylus ear-shaped.

##### Description.

**Male.** Body length 8.0 mm, wing length 9.0 mm.

Vertex and frons black; face and clypeus yellow with brown hairs; gena yellow with a black elliptical spot medially, hairs on gena dark brown; occiput yellow. Compound eyes black without pubescence. Scape, pedicel and basal 1/2 of 1^st^ flagellomere yellow, remaining flagellomeres dark brown; hairs dark brown. Proboscis yellow with brown hairs. Palpus yellow with last segment gradually darked apically, hairs brown.

***Thorax*** (Fig. 1a, b). Pronotum and propleuron yellow. Prescutum mostly brownish black, anterior margin with lateral area yellow; scutum and paratergite mostly brownish black, posterior margin yellow; scutellum mostly brownish black, middle area yellow with a patch of dense brown hairs; postnotum yellow. Mesopleuron uniformly yellow. Coxae and trochanters yellow. Wing (Fig. 2a) 3.3 times as long as wide, subhyaline, apical 1/2 slightly brown, marked with small brown marks at base of Rs, tip of R_1_, base of R_2+3_, fork of R_4+5_, r-m, and fork of M_1+2_. Veins brown; Sc ending in C exceeding basal 1/3 of R_2+3_; Rs straight, 4 times the length of r-m; r-m arise from Rs. Wing with setae below fold in cell cua_2_, and over tip 1/3 of wing. Halter and prehaltere pale yellow with brown hairs.

***Abdomen*.** First tergum yellow with caudal 1/3 brownish black, 2^nd^ tergum brownish black with middle area yellow, 3^rd^ tergum yellow with caudal 1/3 brownish black, 4^th^ tergum yellow with caudal 1/2 black, 5^th^ to 7^th^ terga black; first to 4^th^ sterna yellow, 5^th^ to 7^th^ sterna black; hairs on abdomen yellow.

***Male genitalia*** (Figs 3a, 4a–c, 5a–c) black except caudal 1/2 of epandrial clasper brownish yellow. Epandrium (Fig. 4a) bilobed, epandrial lobe narrow, epandrial clasper tapering and slightly curved distally to the middle, inner side with two papillary projections, with brown long hairs; epiproct V-shaped, with short hairs. Gonocoxite (Fig. 4b) long and stout, 2 times as long as wide, basal apodeme small; apical process of paramere triangular, apex semilunar. Gonostylus (Fig. 4b): anterior lobe of basal lobe of gonostylus elliptic with dense short hairs; medial lobe of basal lobe of gonostylus ear-shaped with dense short hairs; secondary lobe of apical stylus of gonostylus finger-shaped, slightly curved distally with several long hairs; tertiary lobe of apical stylus of gonostylus triangular, pointed apically; apical stylus of gonostylus finger-shaped, swollen distally with long hairs. Hypandrium (Fig. 4c): basal division of hypandrium dumbbell-shaped basally with dense long hairs posteriorly; spathate lobe of hypandrium triangular with several long hairs; lateral extension of terminal division of hypandrium elliptic with dense long hairs on posterior 1/2; terminal division of hypandrium papillary. Aedeagus (Fig. 5a–c): subapical sclerite tongue-shaped, apex of subapical sclerite round; aedeagal sclerites with apex laterally compressed, with dorsal corner extended dorsoanterior, curved sided and convergent; lateral ejaculatory processes with base narrow, extended anterolaterally; sperm sac subspherical; ejaculatory apodeme flag-like, closely associated with aedeagal sclerites, larger than sperm sac, paralleling anterior margin of sperm sac.

**Female.** Unknown.

##### Material examined.

China·1♂, ***holotype***; Yunnan Province, Hekou District, Nanxi Town; 132 m; 22 May 2009; T. Zhang leg.; CAU·1♂, ***paratype***; same collection data as holotype; CAU.

##### Distribution.

China (Yunnan).

##### Etymology.

Specific name *hekouensis* (adjective, feminine) referring to the type locality, Hekou.

##### Remarks.

This new species is similar to *P.wangae* from China but can be separated from the latter by the scutellum mostly brownish black with middle area yellow, the 2^nd^ tergum mostly brownish black with middle area yellow, the epandrial clasper with two papillary projections on inner side, and the medial lobe of basal lobe of gonostylus ear-shaped. In *P.wangae*, the scutellum is uniformly yellow brown, the 2^nd^ tergum is mostly yellow with posterior margin brown, the epandrial clasper does not have papillary projection on inner side, and the medial lobe of basal lobe of gonostylus is semicircular (Kang et al. 2013).

#### ﻿ Ptychoptera longa

Taxon classificationAnimaliaDipteraPtychopteridae

Kang, Gao & Zhang sp. nov.

B314C53B-572B-50CE-BDFF-D78D4E00EEBD

https://zoobank.org/E6BFCB06-E52C-452F-94AF-9C2A725CB967

Figs 1c, d , 2b , 3b , 4d–f , 5d–f

##### Diagnosis.

Mesopleuron mostly brown, except upper 1/2 of anepisternum yellow; wing marked with four brown marks and one brown band; epandrium not bilobed, epandrial lobe rectangle; epandrial clasper finger-shaped, merged basally, basal 1/2 broad and rectangle, apical 1/2 narrowing bilaterally; secondary lobe of apical stylus of gonostylus sickle-shaped; terminal division of hypandrium elliptic.

##### Description.

**Male.** Body length 7.5 mm, wing length 7.5 mm.

Vertex and frons brown; face and clypeus yellow with brown hairs; gena yellow with a black elliptical spot medially, hairs on gena brown; occiput yellow. Compound eyes black without pubescence. Scape and pedicel yellow, flagellomeres light yellow; hairs on antenna brown. Proboscis yellow with brown hairs. Palpus yellow with brown hairs.

***Thorax*** (Fig. 1c, d). Pronotum light brown; propleuron yellow. Prescutum, scutum and paratergite uniformly brown; scutellum mostly brown, middle area yellowish brown; postnotum brown, laterotergite with a patch of dense brown hairs. Mesopleuron mostly brown, except upper ½ of anepisternum yellow. Coxae and trochanters yellow. Wing (Fig. 2b) 3.8 times as long as wide, subhyaline, marked with four brown marks and one brown band as follows: four elliptic brown marks at base of cell R, tip of R_1_, fork of R_4+5_, and fork of M_1+2_; one brown band extending from anterior margin of wing, covering base of R_2+3_ and r-m, to the bend in distal section of CuA_2_. Veins brown; Sc ending in C at level of basal 1/3 of R_2+3_; Rs straight, 2 times the length of r-m; r-m arise from R_4+5_. Wing with setae over Sc and Rs, at and below fold in cell cua_2_, and over tip 1/2 of wing (sparse before forks of R_4+5_ and M_1+2_). Halter and prehaltere pale yellow with light brown hairs.

***Abdomen*.** First tergum brown with basal 1/3 yellow, 2^nd^ tergum brown with middle 1/3 yellow, 3^rd^ tergum yellow with caudal 1/3 brown, 4^th^ to 6^th^ terga brown, 7^th^ tergum brown with posterior margin yellow; first to 3^rd^ sterna yellow, 4^th^ to 6^th^ sterna brown with posterior margin yellow, 7^th^ sternum yellow; hairs on abdomen brown.

***Male genitalia*** (Figs 3b, 4d–f, 5d–f) yellow. Epandrium (Fig. 4d) not bilobed, epandrial lobe rectangle, posterior margin with U-shaped concave; epandrial clasper finger-shaped, merged basally, basal 1/2 broad and rectangle, apical 1/2 narrowing bilaterally, with short brown hairs; epiproct triangular with short hairs. Gonocoxite (Fig. 4e) short and stout, 1.5 times as long as wide, basal apodeme small; apical process of paramere papillary, apex with hooked projection. Gonostylus (Fig. 4e): basal lobe of gonostylus ear-shaped with dense short hairs on inner side; secondary lobe of apical stylus of gonostylus sickle-shaped with short hairs; tertiary lobe of apical stylus of gonostylus triangular, round apically; apical stylus of gonostylus long and slender, finger-shaped with short hairs. Hypandrium (Fig. 4f): basal division of hypandrium trapeziform, anterior margin with V-shaped concave, posterior margin with dense long hairs; membranous window of terminal division circular; terminal division of hypandrium elliptic. Aedeagus (Fig. 5d–f): subapical sclerite rectangular, apex of subapical sclerite slightly concave; aedeagal sclerites with apex laterally compressed, with dorsal corner extended dorsoanterior, curved sided and convergent, base broad; lateral ejaculatory processes with base straight, narrow, extended straight anterolaterally; sperm sac subspherical; ejaculatory apodeme flag-like, closely associated with aedeagal sclerites, larger than sperm sac, paralleling anterior margin of sperm sac.

**Female.** Unknown.

##### Material examined.

China·1♂, ***holotype***; Guizhou Province, Suiyang District, Kuankuoshui National Nature Reserve; 11 Aug. 2010; S. Liu leg.; CAU·1♂, ***paratype***; same collection data as holotype; CAU.

##### Distribution.

China (Guizhou).

##### Etymology.

Specific name from Latin *longa* (adjective, feminine, meaning “long”), referring to the long epandrial clasper.

##### Remarks.

This new species is similar to *P.yankovskiana* from China and Korea but can be separated from the latter by first tergum brown with basal 1/3 yellow, the epandrium not bilobed, and the epandrial claspers merged basally. In *P.yankovskiana*, the first tergum is uniformly dark brown, the epandrium is bilobed and the epandrial claspers is not merged basally (Kang et al. 2019).

#### ﻿ Ptychoptera xiaohuangshana

Taxon classificationAnimaliaDipteraPtychopteridae

Kang, Gao & Zhang sp. nov.

196BA69E-8A25-550E-BE5E-D2A5891C132A

https://zoobank.org/564AC7EB-55BD-46AA-8474-8F99541D9028

Figs 1e, f , 2c , 3c , 4g–i , 5g–i

##### Diagnosis.

Mesopleuron mostly brown, except upper 1/2 of anepisternum yellow; wing marked with three brown marks and two brown bands; epandrial clasper with a curved finger-shaped projection interiorly; anterior lobe of basal lobe of gonostylus nose-shaped; medial lobe of basal lobe of gonostylus toothbrush-shaped.

##### Description.

**Male.** Body length 7.0 mm, wing length 7.0 mm.

Vertex and frons brown; face and clypeus yellow with light brown hairs; gena yellow with a black elliptical spot medially, hairs on gena brown; occiput yellow. Compound eyes black without pubescence. Scape and pedicel yellow, flagellomeres light yellow; hairs on antenna brown. Proboscis light yellow with light yellow hairs. Palpus light yellow with light yellow hairs.

***Thorax*** (Fig. 1e, f). Pronotum and propleuron light brown. Prescutum, scutum, and paratergite uniformly brown; scutellum mostly brown, middle area yellowish brown; postnotum brown, laterotergite with a patch of dense brown hairs. Mesopleuron mostly brown, except upper ½ of anepisternum yellow. Coxae and trochanters yellow; femora yellow with brown ring apically; hairs on legs brown. Wing (Fig. 2c) 3.8 times as long as wide, subhyaline, marked with three brown marks and two brown bands as follows: one triangular brown mark at base of M, two elliptic brown marks at base of Rs and at midlength of CuA_1_; median band extending from anterior margin of wing, covering base of R_2+3_ and r-m, to the bend in distal section of CuA_2_; subapical band extending from anterior margin of wing, covering tip of R_1_, R_2_, and fork of R_4+5_, to fork of M_1+2_.Veins brown; Sc ending in C not at level of basal third of R_2+3_; Rs slightly curved medially, 4.1 times the length of r-m; r-m arise from Rs. Wing with setae over Sc and Rs, at and below fold in cell cua_2_, and over tip 1/2 of wing (slightly sparse before forks of R_4+5_ and M_1+2_). Halter and prehaltere pale yellow with light brown hairs.

***Abdomen*.** First tergum light brown, 2^nd^ tergum light brown with middle 1/3 yellow, 3^rd^ tergum yellow caudal 1/2 light brown, 4^th^ to 6^th^ terga light brown, 7^th^ tergum yellow with basal 1/3 light brown; first to 3^rd^ sterna yellow, 4^th^ to 6^th^ sterna light brown with posterior margin yellow, 7^th^ sternum yellow with basal 1/3 light brown; hairs on abdomen light brown.

***Male genitalia*** (Figs 3c, 4g–i, 5g–i) brown. Epandrium (Fig. 4g) bilobed, epandrial lobe semicircular; epandrial clasper broad basally, with a curved finger-shaped projection interiorly, finger-shaped projection basally narrow, apically swollen, with uniformly long hairs; epandrial clasper narrowed medially and slightly curved ventrally, slightly swollen and flat apically, with dense long hairs; epiproct triangular, with two papillary projections posteriorly, with short hairs. Gonocoxite (Fig. 4h) broad, 2 times as long as wide, inner side with a triangular projection medially, with dense hairs; basal apodeme small; apical process of paramere hooked. Gonostylus (Fig. 4h): anterior lobe of basal lobe of gonostylus nose-shaped with several long hairs; medial lobe of basal lobe of gonostylus toothbrush-shaped, with a hairy semilunar lobe basally and a hairy papillary projection medially; apical stylus of gonostylus finger-shaped with short hairs. Hypandrium (Fig. 4i): basal division of hypandrium triangular, anterior margin with V-shaped concave; terminal division of hypandrium gourd-shaped with dense short hairs. Aedeagus (Fig. 5g–i): subapical sclerite triangular, apex of subapical sclerite flat; aedeagal sclerites with apex laterally compressed, with dorsal corner extended dorsoanterior, curved sided and convergent, base broad; lateral ejaculatory processes with base straight, extended straight anterolaterally; sperm sac subspherical; ejaculatory apodeme flag-like, closely associated with aedeagal sclerites, larger than sperm sac, paralleling anterior margin of sperm sac.

**Female.** Unknown.

##### Material examined.

China·1♂, ***holotype***; Guangdong Province, Ruyuan District, Nanling National Forest Park, Mount Xiaohuangshan; 24 Aug. 2010; T. Zhang leg.; CAU·1♂, ***paratype***; same collection data as holotype; CAU.

##### Distribution.

China (Guangdong).

##### Etymology.

Specific name *xiaohuangshana* (adjective, feminine) referring to the type locality, Mount Xiaohuangshan.

##### Remarks.

This new species is similar to *P.bellula* from China but can be separated from the latter by the 2^nd^ tergum light brown with middle 1/3 yellow, the epandrial clasper with a curved finger-shaped projection interiorly, the epiproct with two papillary projections posteriorly, and the medial lobe of basal lobe of gonostylus toothbrush-shaped. In *P.bellula*, the 2^nd^ tergum is black with base yellow, the epandrial clasper does not have a curved finger-shaped projection interiorly, the epiproct have a strongly haired papillary projection posteriorly, and the medial lobe of basal lobe of gonostylus is semilunar (Alexander 1937; Krzeminski and Zwick 1993).

## Supplementary Material

XML Treatment for Ptychoptera hekouensis

XML Treatment for Ptychoptera longa

XML Treatment for Ptychoptera xiaohuangshana

## References

[B1] Alexander CP (1924) Undescribed species of Japanese Ptychopteridae (Diptera). Insecutor Inscitiae Menstruus 9: 80–83.

[B2] Alexander CP (1935) New or little-known Tipulidae from eastern Asia (Diptera). XXIII–XXVII. Philippine Journal of Science 56: 339–372.

[B3] Alexander CP (1937) New species of Ptychopteridae (Diptera). Bulletin of the Brooklyn Entomological Society 32: 140–143.

[B4] Alexander CP (1945) Undescribed species of crane-flies from northern Korea (Diptera, Tipuloidea). Transactions of the Royal Entomological Society of London 95(4): 227–246. 10.1111/j.1365-2311.1945.tb00261.x

[B5] Alexander CP (1981) Ptychopteridae. In: McAlpine JF Peterson BV Shewell GE Teskey HJ Vockeroth JR Wood DM (Eds) Manual of Nearctic Diptera (Vol.I). Agriculture Canada Monograph 27. Agriculture Canada, Ottawa, 325–328.

[B6] Fasbender A (2014) Phylogeny and diversity of the phantom crane flies (Diptera: Ptychopteridae). PhD Dissertation, Iowa State University, Ames, 855 pp.

[B7] Kang ZH Yao G Yang D (2013) Five new species of *Ptychoptera* Meigen with a key to species from China (Diptera: Ptychopteridae). Zootaxa 3682(4): 541–555. 10.11646/zootaxa.3682.4.5 25243309

[B8] Kang ZH Xue ZX Zhang X (2019) New species and record of *Ptychoptera* Meigen, 1803 (Diptera: Ptychopteridae) from China. Zootaxa 4648(3): 455–4723. 10.11646/zootaxa.4648.3.3 31716935

[B9] Krzeminski W Zwick P (1993) New and little known Ptychopteridae (Diptera) from the Palaearctic region. Aquatic Insects 15(2): 65–87. 10.1080/01650429309361504

[B10] McAlpine JF (1981) Morphology and terminology: Adults. In: McAlpine JF Peterson BV Shewell GE Teskey HJ Vockeroth JR Wood DM (Eds) Manual of Nearctic Diptera Vol.1. Agriculture Canada Monograph 27. Agriculture Canada, Ottawa, 9–63.

[B11] Meigen JW (1803) Versuch einer neuen Gattungs-Eintheilung der europaischen zweiflugligen Insekten. Magazin für Insektenkunde (Illiger) 2: 259–281.

[B12] Nakamura T Saigusa T (2009) Taxonomic study of the family Ptychopteridae of Japan (Diptera). Zoosymposia 3(1): 273–303. 10.11646/zoosymposia.3.1.23

[B13] Rozkošný R (1997) Family Ptychopteridae. In: Papp L Darvas B (Eds) Contributions to a Manual of Palaearctic Diptera (with Special Reference to Flies of Economic Importance), Volume 2: Nematocera and Lower Brachycera.Science Herald, Budapest, 291–297.

[B14] Shao JQ Kang ZH (2021) New species of the genus *Ptychoptera* Meigen, 1803 (Diptera, Ptychopteridae) from Zhejiang, China with an updated key to Chinese species. ZooKeys 1070: 87–99. 10.3897/zookeys.1070.67779 34819773 PMC8602214

[B15] Yang JK (1996) New record of family Ptychopteridae in Xinglongshan (Diptera: Ptychopteridae). In: Wang X (Ed.) Resources Background Investigation of Gansu Xinglongshan National Nature Reserve.Gansu Minorities Press, Gansu, 288–289.

[B16] Yang JK Chen HY (1995) Diptera: Ptychopteridae. In: Zhu T (Ed.) Insects and Macrofungi of Gutianshan, Zhejiang.Zhejiang Scientech Press, Hangzhou, 180–182.

[B17] Yang JK Chen HY (1998) Diptera: Ptychopteridae. In: Wu H (Ed.) Insects of Longwangshan Nature Reserve.China Forestry Publishing House, Beijing, 240–241.

[B18] Zhang X Kang ZH (2021) Two new species of the genus *Ptychoptera* Meigen, 1803 (Diptera, Ptychopteridae) from Yunnan, China with remarks on the distribution of Chinese species. ZooKeys 1070: 73–86. 10.3897/zookeys.1070.58859 34819772 PMC8602213

